# Editorial: Future prospects of learning in the clinical environment: exploring the technological revolution

**DOI:** 10.3389/fmed.2025.1673779

**Published:** 2025-09-09

**Authors:** Hany Atwa, Mohamed Hany Shehata, Asmaa Abdelnasser, Doaa Kamal, Nourhan F. Wasfy, Komal Atta, Enjy Abouzeid

**Affiliations:** ^1^Medical Education Department, College of Medicine and Health Sciences, Arabian Gulf University, Manama, Bahrain; ^2^Medical Education Department, Faculty of Medicine, Suez Canal University, Ismailia, Egypt; ^3^Family and Community Medicine Department, College of Medicine and Health Sciences, Arabian Gulf University, Manama, Bahrain; ^4^Family Medicine Department, Faculty of Medicine, Helwan University, Helwan, Egypt; ^5^Health Professions Education Center, Ibn Sina National College for Medical Studies, Jeddah, Saudi Arabia; ^6^Department of Basic Medical Sciences, Prince Sattam bin Abdulaziz University, Al-Kharj, Saudi Arabia; ^7^Medical Education Department, University Medical & Dental College, University of Faisalabad, Faisalabad, Pakistan; ^8^School of Medicine, Faculty of Life and Health Sciences, University of Ulster, Londonderry, United Kingdom

**Keywords:** clinical learning environment, simulation-based learning, virtual reality (VR) and augmented reality (AR), extended reality (XR), artificial intelligence in medical education, technology-enhanced learning, health professions education, blended and hybrid learning

The clinical learning environment is undergoing a significant transformation driven by an accelerating wave of technological innovation. The landscape of healthcare education is shifting from conventional, didactic approaches to more interactive, immersive, and personalized modalities. As emerging technologies such as virtual reality (VR), augmented reality (AR), artificial intelligence (AI), extended reality (XR), and metaverse-based environments gain traction, educators are reimagining how learning occurs, particularly in clinical contexts where practical skills, decision-making, and empathy are paramount (1).

This Research Topic, “*Future Prospects of Learning in the Clinical Environment: Exploring the Technological Revolution*,” brings together 13 articles that exemplify the diverse and evolving roles of educational technology in shaping the future of clinical training. These 13 articles address five important key themes and technological modalities, as shown in Figure 1. The collective contributions present compelling evidence, insights, and innovations that inform how we design, implement, and evaluate learning in healthcare professions.

**Figure 1:**
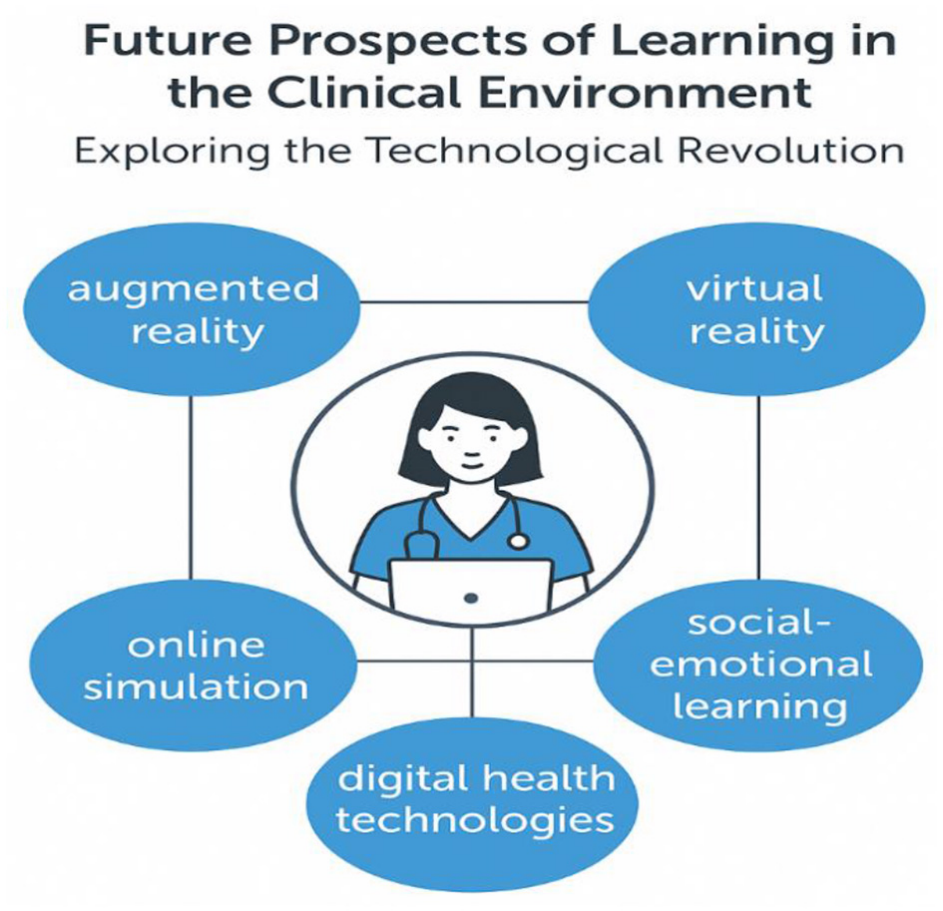
Identified key themes and technological modalities influencing the future of clinical education.

A prominent theme emerging from this Research Topic is the power of simulation-based learning. VR simulation has shown notable promise in nursing education. A systematic review and meta-analysis by Cho and Kim on enhancing nursing competency through virtual reality simulation among nursing students demonstrated its effectiveness in enhancing core competencies, engagement, and clinical confidence. Simulation-based learning aligns with the broader literature on virtual patients and interactive learning tools (2).

Similarly, targeted simulations such as GASMAN software combined with case-based learning (Chen et al.) have proven superior to traditional lecture-based learning for inhalation anesthesia, indicating that interactivity significantly boosts knowledge retention and clinical reasoning.

Blended and hybrid models are also gaining momentum. The BOPPPS (Bridge-In, Objectives, Pretest, Participatory Learning, Post-test, and Summary) teaching model was rigorously evaluated in a meta-analysis by Li Y. et al., revealing its advantages in student satisfaction and academic performance. Other studies explored the merits of combining online and offline instruction. For example, an O2O (online-to-offline) teaching approach for non-anesthesiology residents in anesthesiology (Zhao et al.) and a blended model in clinical laboratory hematology (Li D. et al.) both underscored increased engagement, deeper learning, and practical skill acquisition (3).

Technological integration is not limited to hardware and platforms; it also challenges us to reimagine our pedagogical frameworks. A novel conceptual model of technology-enhanced practice competencies (Perle et al.) illustrates how competencies in digital literacy, ethics, and data management are becoming indispensable in clinical practice (4).

Beyond competencies, these technologies are reshaping fundamental educational theories. The shift from didactic delivery to immersive, interactive platforms aligns with constructivist and experiential learning paradigms, where knowledge is co-constructed through active engagement. Likewise, assessment practices are evolving to include performance-based tasks, real-time analytics, and AI-driven feedback, reflecting a broader redefinition of what constitutes competence in the clinical learning environment.

The metaverse, a once futuristic concept, is now being considered as a transformative tool in nursing practice and education. In their review, Li X. et al. present how immersive digital spaces can be leveraged for telemedicine, surgical assistance, chronic disease management, and psychological support. By merging physical and digital realities, the metaverse creates new spatial and relational dimensions for healthcare delivery and learning.

Meanwhile, extended reality (XR) technologies are making waves globally. A cross-sectional study in Pakistan by Khan et al. highlighted the enthusiasm among healthcare professionals and students toward XR technology, despite infrastructural and regulatory challenges. It emphasized the need for strategic planning to integrate XR into national healthcare education systems.

Simulation fidelity and realism continue to be central to clinical competence. An innovative Italian study by Neri et al. employed SimLife^®^ to create hyper-realistic scenarios in medical training, providing students with a closer approximation to real-life patient interaction and physiological response.

Airway management, a critical component in clinical practice, was targeted in a scenario-based teaching intervention (Lin et al.). Results revealed enhanced procedural knowledge and confidence among anesthesia undergraduates, validating the potential of scenario-based online platforms in bridging theoretical and procedural gaps (5).

Notably, technology is also fostering new forms of reflective and emotional engagement. Social-emotional learning (SEL) is proposed as an essential complement to technological skills in medical training (Hsu et al.). SEL strengthens empathy, resilience, and professionalism—traits that are foundational for holistic medical care but are at risk of being overlooked in tech-centric models.

Even audio-based educational tools are under scrutiny. A critical analysis of cardiology podcasts by Kamalanathan et al. pointed out deficiencies in content transparency and referencing, urging the need for quality control standards in this increasingly popular modality.

Artificial intelligence is reshaping not just the delivery of care but also the way we teach and explain it—prompting educators to rethink sources of clinical knowledge.

While these studies provide promising evidence for the integration of simulation, blended models, XR, and metaverse-based learning, it is important to acknowledge their methodological limitations. Many rely on small samples, self-reported outcomes, or short-term assessments. In particular, research on VR and XR often carries inherent biases linked to novelty effects and limited generalizability across institutions and resource settings. A cautious interpretation is therefore essential, underscoring the need for more longitudinal, multicenter investigations.

Collectively, the studies articulate a vision of a technologically enhanced, learner-centered future where the clinical learning environment is deeply intertwined with technology. However, with opportunity comes responsibility. Equity in access, ethical deployment, standardization of content, and emotional intelligence must be cornerstones of this transformation.

## Conclusion

The technological revolution is not merely altering educational tools—it is transforming how we think, teach, and learn. As we navigate this pivotal era, we must ensure that technology serves pedagogy, not the other way around. Future research and policy should not only document immediate learning gains but also measure the sustained impact of these technologies on graduate competence, patient outcomes, and healthcare system performance. Embedding evaluation frameworks that track long-term educational and clinical effects will be crucial in determining whether these innovations translate into tangible benefits for patients and societies. This Research Topic reflects a vibrant, multidisciplinary effort to imagine new possibilities for clinical education. Our challenge now is to move from innovation to integration, ensuring that digital advancements translate into meaningful, equitable, and human-centered learning experiences for all healthcare professionals.
